# Confusions in orbivirus protein classification

**DOI:** 10.1186/1743-422X-9-166

**Published:** 2012-08-21

**Authors:** Meik Dilcher, Manfred Weidmann

**Affiliations:** 1 Department of Virology, University Medical Center Göttingen, Kreuzbergring 57, D-37075, Göttingen, Germany

**Keywords:** *Reoviridae*, *Orbivirus*, Protein classification

## Abstract

An extensive comparative analysis of orbivirus genomes revealed four cases of unclear numeration and protein designation, due to confused reference to protein size or segment size by which they are encoded. A concise nomenclature based on type species, sequence homology and functional characteristics independent of segment or protein size is suggested.

## Background

The genus *Orbivirus* is one of 15 in the family of *Reoviridae* containing 22 serogroups (species) and at least 160 different serotypes (strains) [1]. Orbiviruses are transmitted by insects (midges, flies, mosquitoes) or by ticks. Their double-stranded RNA (dsRNA) genomes consist of 10 segments coding for seven structural and at least three non-structural proteins. Orbiviruses have no envelope but a double-shelled icosahedral capsid [2] and include pathogenic agents of wild animals (Epizootic hemorrhagic disease virus (EHDV)), domestic animals (Bluetongue virus (BTV) and African horse sickness virus (AHSV)), and of man (Kemerovo virus (KEMV)) [3]. Type species of the genus is the *Culicoides* midge transmitted BTV. Insect-borne orbiviruses are much better characterized than tick-transmitted orbiviruses for which few sequences have been described: Broadhaven virus (BRDV, partial) [4], Sandy Bay virus (SBaV, partial (formerly Nugget virus)) [5-8], St Croix River virus (SCRV, complete genome) [9], Great Island virus (GIV, complete genome) [7].

Recently we determined the complete genomes of Tribeč virus (TRBV) and KEMV in a pyrosequencing approach [10] complementing available partial information on segments 1, 2 and 6 of these viruses and of Lipovnik virus (LIPV) [7].

During our extensive comparative analysis of orbivirus genomes we noticed four cases of unclear numeration and protein designation (see Tables 1 and 2). Some laboratories classify orbivirus proteins according to the size of the proteins whereas others use the size of genome segments from which they are encoded.

(i) The inner shell protein T2 for example can be encoded by segment 2 (tick- and mosquito-borne orbiviruses) or segment 3 (C*ulicoides*-borne orbiviruses). This leads to some laboratories labeling this protein VP2(T2) (e.g. GIV), while others designate it VP3(T2) as in the type species BTV. For Peruvian horse sickness virus (PHSV) however, the segment 2 encoded protein is designated VP3(T2) although it is larger (925 amino acids) than the segment 3 encoded VP2 protein (881 amino acids) [11]. To avoid confusion with the outer shell protein VP2 we suggest to exclusively use VP3(T2) for all T2 proteins.

(ii) VP2 and VP2 homologous proteins can be encoded by segments 2, 3, 4 and 5 and are designated VP2, VP3 (YUOV, SCRV) or VP4 (BRDV segment 4 ( [12,13], sequence entry to GenBank missing)), GIV segment 5). Because of the location on the outer capsid and the described sequence similarity with other VP2 proteins, we suggest that the VP4 proteins (BRDV, GIV) as well as the VP3 proteins (YUOV, SCRV) should be uniformly termed VP2, even though tick-borne VP2 proteins have only half the size of insect-borne VP2 proteins [13].

(iii) The capping enzyme VP4(CaP) can be encoded by segment 4 (BTV, YUOV, SCRV etc.) or segment 3 (TRBV, KEMV). In GIV this protein is designated VP3(CaP) [7] and should be renamed VP4(CaP) to avoid confusions with VP3(T2).

(iv) In most cases VP5 is encoded by segment 6 and comprises a component of the outer shell that might be involved in membrane fusion and penetration [14]. TRBV and KEMV also encode VP5 on segment 6. The highest similarity of TRBV VP5 is to LIPV VP5 (95.6%), again encoded by segment 6 [7]. However, VP5 of BRDV is described as encoded by segment 5 [15]. Since in the classification of the viral genome segments bigger segments have smaller segment numbers, and the size of BRDV segment 6 (1714 bp) encoding the NS1(TuP) [16] is larger than the size of BRDV segment 5 (1658 bp) encoding VP5, a reassignment of BRDV segment 5 and 6 (a vice versa switch) seems necessary.

**Table 1:** Comparison of the genome segments and encoded proteins of BTV, YUOV, TRBV, KEMV and GIV

**BTV (insect-transmitted)**				**YUOV (insect-transmitted)**				**TRBV (tick-transmitted)**				**KEMV (tick-transmitted)**				**GIV (tick-transmitted)**			
Segment 1	VP1 (Pol)	150 kDa	RNA-dep.-RNA- Polymerase	Segment 1	VP1 (Pol)	151 kDa	RNA-dep.-RNA- Polymerase	Segment 1	VP1 (Pol)	146 kDa	RNA-dep.-RNA- Polymerase	Segment 1	VP1 (Pol)	146 kDa	RNA-dep.-RNA- Polymerase	Segment 1	VP1 (Pol)	147 kDa	RNA-dep.-RNA- Polymerase
3944 bp	ACR58458	1302 AA		3393 bp	YP_443925	1315 AA		3892 bp	HQ266581	1284 AA		3896 bp	HQ266591	1285 AA		3897 bp	ADM88592	1285 AA	

Segment 2	**VP2**	111 kDa	**Outer shell**	Segment 2	**VP2 (T2)**	107 kDa	**Inner shell**	Segment 2	**VP3 (T2)**	102 kDa	**Inner shell**	Segment 2	**VP3 (T2)**	103 kDa	**Inner shell**	Segment 2	**VP2 (T2)**	103 kDa	**Inner shell**
2953 bp	ACR58459	956 AA		2900 bp	YP_443926	940 AA		2793 bp	HQ266582	908 AA		2792 bp	HQ266592	908 AA		2794 bp	ADM88593	908 AA	

Segment 3	**VP3 (T2)**	103 kDa	**Inner shell**	Segment 3	**VP3**	100 kDa	**Outer shell**	Segment 3	VP4 (CaP)	72 kDa	Capping Enzyme	Segment 3	VP4 (CaP)	72 kDa	Capping Enzyme	Segment 3	VP3 (CaP)	73 kDa	Capping Enzyme
2772 bp	ACR58460	901 AA		2688 bp	YP_443927	873 AA		1935 bp	HQ266583	628 AA		1934 bp	HQ266593	632 AA		1936 bp	ADM88594	635 AA	

Segment 4	VP4 (CaP)	75 kDa	Capping Enzyme	Segment 4	VP4 (CaP)	74 kDa	Capping Enzyme	Segment 4	NS1 (TuP)	62 kDa	Formes Tubules	Segment 4	**VP2**	63 kDa	**Outer shell**	Segment 4	NS1 (TuP)	60 kDa	Formes Tubules
1980 bp	ACR58461	644 AA		1993 bp	YP_443928	645 AA		1734 bp	HQ266584	529 AA		1730 bp	HQ266594	554 AA		1731 bp	ADM88595	531 AA	

Segment 5	NS1 (TuP)	64 kDa	Forms Tubules	Segment 5	NS1 (TuP)	67 kDa	Forms Tubules	Segment 5	**VP2**	62 kDa	**Outer shell**	Segment 5	NS1 (TuP)	60 kDa	Formes Tubules	Segment 5	**VP4**	62 kDa	**Outer shell**
1769 bp	ACR58463	552 AA		1957 bp	YP_443929	574 AA		1730 bp	HQ266585	554 AA		1719 bp	HQ266595	529 AA		1722 bp	ADM88596	551 AA	

Segment 6	**VP5**	59 kDa	**Outer shell**	Segment 6	**VP5**	59 kDa	**Outer shell**	Segment 6	**VP5**	59 kDa	**Outer shell**	Segment 6	**VP5**	59 kDa	**Outer shell**	Segment 6	**VP5**	60 kDa	**Outer shell**
1638 bp	ACR58462	526 AA		1683 bp	YP_443930	535 AA		1668 bp	HQ266586	537 AA		1668 bp	HQ266596	537 AA		1666 bp	ADM88597	537 AA	

Segment 7	**VP7 (T13)**	39 kDa	**Inner shell**	Segment 7	NS2 (ViP)	48 kDa	Viral inclusion body matrix protein	Segment 7	NS2 (ViP)	41 kDa	Viral inclusion body matrix protein	Segment 7	NS2 (ViP)	41 kDa	Viral inclusion body matrix protein	Segment 7	**VP7 (T13)**	40 kDa	**Inner shell**
1156 bp	ACR58464	349 AA		1504 bp	YP_443931	435 AA		1196 bp	HQ266587	368 AA		1197 bp	HQ266597	368 AA		1181 bp	ADM88598	357 AA	

Segment 8	NS2 (ViP)	41 kDa	Viral inclusion body matrix protein	Segment 8	**VP7 (T13)**	40 kDa	**Inner shell**	Segment 8	**VP7 (T13)**	40 kDa	**Inner shell**	Segment 8	**VP7 (T13)**	40 kDa	**Inner shell**	Segment 8	NS2 (ViP)	39 kDa	Viral inclusion body matrix protein
1125 bp	ACR58465	354 AA		1191 bp	YP_443932	355 AA		1184 bp	HQ266588	357 AA		1183 bp	HQ266598	357 AA		1172 bp	ADM88599	359 AA	

Segment 9	VP6 (Hel)	36 kDa	ssRNA and dsRNA binding helicase	Segment 9	VP6 (Hel)	37 kDa	ssRNA and dsRNA binding helicase	Segment 9	VP6 (Hel)	33 kDa	ssRNA and dsRNA binding helicase	Segment 9	VP6 (Hel)	34 kDa	ssRNA and dsRNA binding helicase	Segment 9	VP6 (Hel)	34 kDa	ssRNA and dsRNA binding helicase
1049 bp	ACR58466	329 AA		1082 bp	YP_443933	338 AA		1034 bp	HQ266589	312 AA		1049 bp	HQ266599	317 AA		1056 bp	AMD88600	321 AA	

Segment 10	NS3	26 kDa	Glycoprotein	Segment 10	NS3	28 kDa	Glycoprotein	Segment 10	NS3	23 kDa	Glycoprotein	Segment 10	NS3	23 kDA	Glycoprotein	Segment 10	NS3	19 kDa	Glycoprotein
822 bp	ACR58467	229 AA		825 bp	YP_443934	253 AA		705 bp	HQ266590	214 AA		707 bp	HQ266600	214AA		703 bp	ADM88602	171 AA	

Outer and inner shell proteins are labeled in bold. GenBank and SwissProt accession numbers are indicated.

**Table 2:** Comparison of the genome segments and encoded proteins of SCRV, PHSV, BRDV and LIPV

**SCRV (tick-transmitted)**				**PHSV (isolates only known from horses)**				**BRDV (tick-transmitted)**				**LIPV (tick-transmitted)**			
Segment 1	VP1 (Pol)	151 kDa	RNA-dep.-RNA-Polymerase	Segment 1	VP1 (Pol)	151 kDa	RNA-dep.-RNA-Polymerase	Segment 1				Segment 1	VP1 (Pol)	146 kDa	RNA-dep.-RNA-Polymerase
4089 bp	YP_052942	1345 AA		3987 bp	YP_460038	1311 AA						3892 bp	ADM88603	1284 AA	

Segment 2	**VP2 (T2)**	98 kDa	**Inner shell**	Segment 2	**VP3 (T2)**	105 kDa	**Inner shell**	Segment 2	**VP2 (T2)**	103 kDa	**Inner shell**	Segment 2	**VP2 (T2)**	103 kDa	**Inner shell**
2747 bp	YP_052943	890 AA		2856 bp	YP_460039	925 AA			P35934	908 AA		2793 bp	ADM88604	908 AA	

Segment 3	**VP3**	74 kDa	**Outer shell**	Segment 3	**VP2**	104 kDa	**Outer shell**	Segment 3				Segment 3			
2024 bp	YP_052944	654 AA		2747 bp	YP_460040	881 AA									

Segment 4	VP4 (CaP)	74 kDa	Capping Enzyme	Segment 4	VP4 (CaP)	74 kDa	Capping Enzyme	Segment 4	**VP4** †	63 kDa †	**Outer shell**	Segment 4			
2017 bp	YP_052945	643 AA		1996 bp	YP_460041	646 AA									

Segment 5	**VP5**	57 kDa	**Outer shell**	Segment 5	NS1 (TuP)	64 kDa	Forms Tubules	Segment 5	**VP5**	53 kDa	**Outer shell**	Segment 5			
1664 bp	YP_052946	517 AA		1784 bp	YP_460045	554 AA		1658 bp	P21230	480 AA					

Segment 6	NS1 (TuP)	58 kDa	Forms Tubules	Segment 6	**VP5**	59 kDa	**Outer shell**	Segment 6	NS1 (TuP)	60 kDa	Formes Tubules	Segment 6	**VP5**	502 AA ††	**Outer shell**
1657 bp	YP_052947	517 AA		1695 bp	YP_460042	529 AA		1714 bp	2115436A	537 AA		1509 bp ††	ADM88605		

Segment 7	NS2 (ViP)	51 kDa	Viral inclusion body matrix protein	Segment 7	NS2 (ViP)	48 kDa	Viral inclusion body matrix protein	Segment 7	**VP7 (T13)**	40 kDa	**Inner shell**	Segment 7			
1463 bp	YP_052948	462 AA		1613 bp	YP_460046	435 AA			P35935	356 AA					

Segment 8	**VP7 (T13)**	41 kDa	**Inner shell**	Segment 8	**VP7 (T13)**	40 kDa	**Inner shell**	Segment 8				Segment 8			
1256 bp	YP_052949	379 AA		1180 bp	YP_460044	353 AA									

Segment 9	VP6 (Hel)	26 kDa	ssRNA and dsRNA binding helicase	Segment 9	VP6 (Hel)	37 kDa	ssRNA and dsRNA binding helicase	Segment 9				Segment 9			
764 bp	YP_052950	232 AA		1071 bp	YP_460043	334 AA									

Segment 10	NS3	24 kDa	Glycoprotein	Segment 10	NS3	28 kDa	Glycoprotein	Segment 10	NS3	22 kDa	Glycoprotein	Segment 10			
764 bp	YP_052951	224 AA		819 bp	YP_460047	255 AA			P32555	205 AA					

Outer and inner shell proteins are labeled in bold. GenBank and SwissProt accession numbers are indicated.

†: [12,13], GenBank entry missing.

††: partial sequence.

To summarize, it would be much more helpful if the nomenclature of the viral proteins in orbiviruses would reflect the sequence homology and functional relationship rather than protein size or encoding segment size, since the sizes of the orbivirus genome segments sometimes only differ slightly, which leads to even closely related viruses such as TRBV and KEMV encoding VP2 and NS1(TuP) on different genome segments. We therefore suggest the following concise nomenclature based on the type species BTV and on sequence homology and functional characteristics independent of segment or protein size: VP1(Pol), VP2, VP3(T2), VP4(CaP), VP5, VP6(Hel), VP7(T13), NS1(TuP), NS2(ViP), NS3.

## Competing interests

The authors declare that they have no competing interests.

## Author’s contributions

MD and MW wrote the paper. Both authors read and approved the final manuscript.
